# Colon cancer risk and different HRT formulations: a case-control study

**DOI:** 10.1186/1471-2407-7-76

**Published:** 2007-05-08

**Authors:** Jürgen C Dinger, Lothar AJ Heinemann, Sabine Möhner, Do Minh Thai, Anita Assmann

**Affiliations:** 1 Centre for Epidemiology & Health Research Berlin, Invalidenstr. 115, 10115 Berlin, Germany

## Abstract

**Background:**

Most studies have found no increased risk of colon cancer associated with hormone replacement therapy (HRT), or even a decreased risk. But information about the effects of different HRT preparations is lacking.

**Methods:**

A case-control study was performed within Germany in collaboration with regional cancer registries and tumor centers. Up to 5 controls were matched to each case of colon cancer. Conditional logistic regression analysis was applied to estimate crude and adjusted odds ratios (OR) and 95% confidence intervals (95% CI). Stratified analyses were performed to get an impression of the risk associated with different estrogens and progestins.

**Results:**

A total of 354 cases of colon cancer were compared with 1422 matched controls. The adjusted overall risk estimate for colon cancer (ColC) associated with ever-use of HRT was 0.97 (0.71 – 1.32). No clinically relevant trends for ColC risk were observed with increasing duration of HRT use, or increasing time since first or last HRT use in aggregate.

Whereas the overall risk estimates were stable, the numbers in many of the sub-analyses of HRT preparation groups (estrogens and progestins) were too small for conclusions. Nevertheless, if the ColC risk estimates are taken at face value, most seemed to be reduced compared with never-use of HRT, but did not vary much across HRT formulation subgroups. In particular, no substantial difference in ColC risk was observed between HRT-containing conjugated equine estrogens (CEE) or medroxyprogesterone acetate (MPA) and other formulations more common in Europe.

**Conclusion:**

Ever-use of HRT was not associated with an increased risk of colon cancer. In contrary, most risk estimates pointed non-significantly toward a lower ColC risk in HRT ever user. They did not vary markedly among different HRT formulations (estrogens, progestins). However, the small numbers and the overlapping nature of the subgroups suggest cautious interpretation.

## Background

Complex discussions surrounding the role of steroid hormone formulations, particularly concerning Hormone Replacement Therapy (HRT), experienced a revival after the publication of the Women's Health Initiative (WHI) Study in 2002 [1].

Many earlier studies reported no significant increase in colon cancer for ever-use of HRT, and more often a decreased risk [2,3]. The two arms of the WHI studies [1,4] reported a risk of colon cancer associated with HRT use around unity, and non-significantly reduced, respectively. The HERS studies [5] reported similar findings. However, these results are based on preparations with CEE (conjugated equine estrogens) or CEE plus MPA (medroxyprogesterone acetate) and almost nothing is known about the colon cancer risk associated with different HRT preparations and ways of application.

The primary aim of this publication is to present data on colon cancer risk associated with use of different HRT formulations and administrations.

## Methods

The study method has been recently described in greater detail elsewhere [6]. In brief: The objective of this study is to analyze possible differences in the risk of colon cancer (ColC) associated with different HRT formulations, using a case-control design, i.e. in collaboration with German cancer registries and tumor centers (see acknowledgements).

The lifetime history of exposure to sex steroid hormones was recorded by month and year of exposure (type, brand name). This information was compiled primarily on the basis of a questionnaire. Relevant approvals of the study were obtained (Ethical Committees; Office of Data Privacy).

Eligible cases were histologically confirmed malignant neoplasms of the colon (ICD 10: C18; in accordance with the definition of the IARC Cancer Registry [7]) diagnosed mainly between 2000 and 2004 in women of all age groups. Subjects had to be alive, in sufficiently good health, and willing to complete the questionnaire.

A potential total of 354 cases was included in the analysis. Up to five controls were matched for each ColC case, using the same age (year of birth +/- two years) and residency (Bundesland) as matching criteria. A pool of potential controls from a large cohort study was used – as described in a previous publication 6. We found 1422 matched controls for 354 ColC cases.

### Data collection, variables and database preparation

Time-related information on lifetime history of hormone use as well as data on reproductive life, lifestyle pattern, conditions/diseases, and some other factors were obtained via a self-administered postal questionnaire.

HRT preparations ever used were classified according to the route of administration (oral, transdermal, other), type of combination (sequential, continuous-combined), estrogen type (estradiol, and conjugated equine estrogens (CEE)), and progestin type (Noretisterone acetate (NETA), Levonorgestrel (LNG), Medroxyprogesterone acetate (MPA), Medrogestone, and Tibolone); the numbers for other progestins were too small for calculating risk estimates).

Each participant who had ever used HRT in more than one of the above HRT categories was counted more than once across the analyses (depending on switching pattern), i.e. the categories are *not*mutually exclusive. Therefore, comparison of risk estimates across exposure groups can be used only as a first impression as to whether there are substantial differences or not. We also refrained from defining categories of HRTs in terms of "longest used during lifetime", because of the arbitrary character of the decision as well as the assumed incompatibility for what "longest" might mean across different compounds.

### Index dates

We excluded stepwise information on exposure close to the date of diagnosis, because we cannot exclude the possibility that prior to the final diagnosis of colon cancer, the women and/or their physicians might have been aware of "warning signs" (e.g., history of polyps) that may have led to a decision to start HRT or not previously. A series of index dates was introduced, such as 0.5, 2, 4, and 6 years, i.e. all exposure-related information between these dates and the date of diagnosis was ignored in the respective analyses. We intended to estimate the possible magnitude of such a bias if it exists. In this report, we focus on a lag-time of 0.5 year prior to diagnosis in order to avoid the effects of decisions related to a perceived ColC risk in the time immediately preceding the cancer diagnosis.

### The analytic model

Conditional logistic regression was used as primary analysis. Crude and adjusted odds ratios (OR) were reported with 95% confidence intervals (95% CI). Adjustment variables were BMI, family history of colon cancer, child-bearing history, age at first live birth, duration of breast-feeding, age at menarche, OC use, and education, whereby missing values were imputed (modal value) to allow the analysis. For sub-group analyses of HRT categories, we additionally adjusted for age and residency because the matching effect was lost in the individual subgroups. We were not able to use age at menopause as a co-variable because it could not be reliably determined for a high proportion of participants. Moreover, we refrained from complex adjustment in these subgroup analyses to prevent unstable risk estimates. For the same reason, we did not calculate risk estimates if any cell of the "two-by-two" table contained fewer than 5 women.

We analyzed the impact of duration of HRT as well as time since first and last HRT use for each of four categories. The statistical packages SAS 9.1 and STATA 8.0 were used.

## Results

### Case/non-case characteristics

Table 1 compares the colon cancer cases with the matched controls concerning a list of risk markers and potential confounders. The mean ages of the cancer cases and matched controls were similar (matching).

However, increasing age was significantly associated with increased risk of colon cancer, i.e. both as a continuous (OR = 1.64 per year) and a dichotomized categorical variable (OR = 4.38), as was first-degree family history of colon cancer (OR = 3.40). Significantly reduced ColC risk was observed for higher education (OR = 0.64).

Higher number of children (OR = 0.75 per child), ever breast-feeding (OR = 0.31), and ever-use of oral contraceptives (OR = 0.64) were apparently associated with lower ColC risk. However, the association with HRT use was marginal only, i.e. the effect as confounder negligible.

The result for BMI showed a slight decrease with increasing BMI (marginally significant in the analysis as continuous variable). All other factors listed in Table 1 showed no statistically significant decreased or increased risk.

All the above variables were significant effect modifiers of the association between HRT exposure and ColC risk (data not shown) and therefore included in the analyses to control for confounding, but had a marginal effect only (compare crude and adjusted ORs in the following tables).

### Ever vs. never-use of HRT

Table 2 presents no increased risk of colon cancer in HRT users compared with never users (adj. OR 0.97; 95% confidence interval 0.71 to 1.30) at the index date of 0.5 year. The risk estimates did not vary markedly across all other index dates (data not shown in table 2) and were very similar with and without adjustment.

### Stratified analyses

Table 2 also shows results of the analyses stratified by duration of HRT use, time since first HRT use and time elapsed since last HRT use. Neither longer duration of HRT use nor longer time since first or last use showed meaningful differences in colon cancer risk when analyzed in aggregate, i.e. not considering specific HRT subgroups. Most risk estimates were below unity (1.0) but without statistical significance (however the statistical power of these sub-analyses was low in some subgroups). A significant trend of ColC risk with increasing time was not observed.

### Cancer risk across different HRT formulations

Table 3 provides ColC risk estimates (and 95% confidence intervals) for the index date of 0.5 year by HRT formulations. In addition, the table is stratified by duration of use – to the extent numbers permitted ("rule of 5 per cell" – see methods section). It must be stressed again that the HRT categories are not mutually exclusive, except the three categories of CEE/MPA combination but here again with small numbers of exposed cases.

Ever-use of HRTs via oral and transdermal routes of administration showed no significant association with colon cancer in aggregate, but there was a significantly increasing trend with longer duration of use. The latter was not observed for the other routes of administration but the numbers were tiny.

Both sequential and continuous combined formulations showed virtually identical, non-significantly reduced ColC risk estimates in aggregate. An increasing trend of risk with increasing duration of use was found for sequential formulations but not for continuous-combined, although the confidence intervals were largely overlapping and the numbers in the strata for duration small in continuous-combined HRTs. This was similar for the respective subgroups of duration of use, taking the overlapping confidence intervals into account. This was also true for all estrogen + progestin combinations together.

The seemingly increasing risk estimates with longer duration of use in the subgroup of sequential formulations, for example, showed largely overlapping confidence intervals, as can also be seen for other trends with increasing duration of use.

No obvious or meaningful difference in risk for ColC was found between ever-use of CEE and ever-use of MPA; however, the numbers were too small for the fixed combination of CEE plus MPA.

No meaningful difference in ColC risk was observed between ever-use of estrogen and ever-use of progestin-only formulations, although the numbers for progestin only were too small for meaningful analysis.

A risk of ColC was not significantly associated with either of the two analyzable estrogens, i.e. it was similar and at least not increased (largely overlapping confidence intervals).

Of the progestins, only NETA, LNG, MPA, Medrogestone, and to some extend also Tibolone showed mainly non-significantly decreased ColC risk estimates if taken at face value. Many of the subgroups for duration of use were too small to permit meaningful calculations.

## Discussion

Colorectal cancers altogether are the fourth most common cancer worldwide and commonly thought to be associated with food and nutrition – except for genetic risk and polyposis – as recently expressed by an expert committee from the American Institute for Cancer [8].

We observed other potential risk markers for colon cancer beyond food and nutrition in our case-control study (see table 1). The observation that increasing age as well as family history significantly increases the risk of colon cancer is not new and is shared by the literature. But less common is the observation that many variables associated with reproductive history reduce the ColC risk, such as higher number of children, breast-feeding, and a history of OC use. And these markers are considered to be associated with sex-steroid hormone use, i.e. included in the analysis as confounding variables.

We abstain from a detailed discussion of the impact of above variables of reproductive history on lower risk of ColC, because it was not the objective of our study to answer such questions with relative small numbers and other limitations of the study (see later). Like OC ever-use, information on reproductive history was obtained in a search for differences between cases and controls that possibly could explain different risk of HRT user (potential confounding). Although OC ever-use is closer to the research question of an effect of HRT on colon cancer risk, the study was not designed to analyse the impact of OCs on ColC. Fortunately – taken at face-value – the point estimates of OC use and HRT use (irrespective of formulation) point in a similar direction, i.e. toward lower risk of colon cancer, but mainly non-significant.

The above-mentioned expert panel from the American Institute for Cancer did not assign external sex-steroid hormones a convincing role among important risk factors [8]. On the other hand, the possibility that sex-steroid hormones might play a role cannot be ignored and are debated for years. Estrogen receptors are present in the colon and estrogen-mediated changes to the receptor could be compatible with a decrease in ColC risk. Methylation of DNA is a hot issue in colon cancer genesis and can be discussed as biological plausibility of reduced ColC risk associated with hormone (estrogen) use [9,10].

Most but not all of the previously published epidemiological studies on colon cancer in women found a reduced risk associated with external use of sex-steroid hormones [11]. A review by Franceschi and La Vecchia [2] reported that an inverse association between colon cancer and HRT use was described in 5 of 12 case-control studies with a 20–40% risk reduction; the remaining studies also observed inverse but non-significant associations with HRT use – like in our case-control study. Other cohort studies showed relative risks around or below unity. The information concerning the effect of duration of use and other time-dependent variables differed across studies – if reported at all. In a review and meta-analysis of 18 epidemiological studies, Grodstein [3] found a 20% reduction in ColC risk associated with postmenopausal HRT use (compared with never-use). The discussion suggested that observed differences could be due to different user patterns or types of HRT or just a chance finding notwithstanding the often discussed problem of statistical significance and clinical relevance. The authors of this review stressed that biological evidence supports such an inverse association and found it similar to the observations made regarding oral contraceptives. Even clinical trials have recently reported no or a non-significantly reduced risk of colon cancer, namely the HERS-studies (HR = 0.81) [5] and the WHI studies (HR = 0.63 ns in E+P arm; HR = 1.08 ns in E-only arm) [1,4]. These findings are compatible with the results of our observational study: Overall, non-significant association around or below unity between colon cancer and HRT use compared with never-use. We found a non-significant reduction in the risk of ColC for most HRT subgroups. The small numbers for subgroups of HRT formulations and the subsequently large confidence intervals preclude strong conclusions.

The distribution of ColC risk estimates for different HRT formulation categories is compatible with the notion that no obvious differences were observed. All point estimates were non-significantly around or below unity (1.0). Taken at face value, some point estimates for HRT use would be compatible with a decreased risk of colon cancer. The largely overlapping confidence intervals across various HRT formulation groups argue strongly against convincing differences. The interpretation of the statistically significant trend of increasing risk estimates with increasing duration of use in some but not all HRT subgroups – in contrast to no significant risk trend of duration of HRT use in aggregate – is not clear. This might be a biased or random observation or real, important or clinically meaningless. At least we cannot explain this finding and would hesitate to draw any clinical conclusions from it unless it is confirmed in other studies, i.e. particularly in absence of evidence for an increased ColC risk.

### Limitations of the study

It is a shortcoming of our study that we had small numbers of cases in many of the interesting sub-analyses, and thereby were not able to detect significant differences in colon cancer risk according to different hormone preparations or ways of administration.

Analysis of the effect of external hormones on colon cancer must take into account the lag time in cancer development, although it is difficult for observational studies to account sufficiently for time-dependence. Lag time is likely to be long and may vary depending on complex unknown causal mechanisms, and is therefore an issue involving complex, time-dependent risk factors. Moreover, even observational studies with "state-of-the-art" performance face an important methodological problem if the observed risk estimates are small. Given the great potential for residual confounding and different forms of bias, even a statistically significant small positive or inverse association cannot be ruled out and the results might be inconclusive. Such small associations could well be situated below reliable resolution levels of the "epidemiological microscope", i.e. it is not possible to differentiate between causation and bias/confounding [12,13].

We assume that the results of our study have not been substantially affected by differential surveillance or prevention bias, self-selection or recall bias, although we cannot exclude these possibilities. Another potential source of bias is diagnostic suspicion bias. Nobody can be sure that tumor cases are equally identified (diagnosed) among HRT users and non-users. This, however, would not explain the low point estimates of ColC risk observed, i.e. this would be a conservative estimate.

Despite some advantages of our study over many others in considering time-dependent exposure during the lifetime, or in introducing different lag times to perform stratified analyses, we do not know the impact of the above-mentioned biases on the observed risk estimates. Therefore, our interpretation of the ColC risk estimates observed in the case-control study should be taken with care.

## Conclusion

Our study together with the available literature supports the notion that use of HRT is not associated with an increased risk or even the possibility of an inverse association between colon cancer and HRT use. Obviously, the observed risk did not vary markedly among different HRT formulations (estrogens, progestins). However, the small numbers and the overlapping nature of some of the subgroups suggest cautious interpretation. In addition, there might be differences that were not detectable in our study due to the "limited resolution of the epidemiological microscope". And finally, residual confounding and bias cannot be ruled out.

## Competing interests

This study was funded by the authors' institution. No conflict of interest is declared.

## Authors' contributions

JCD: contributed to the design the study and revision of the manuscript, and was involved in the data analysis; LAJH: responsible for the design of the study and prepared the data analysis plan, responsible for writing the manuscript; SM: responsible for collaboration with the tumor centers as well as quality checks and management of the initial database, contributed to the manuscript; DMT: responsible for final data management and data analysis; contributed to the manuscript; AA: responsible for development of all questionnaires (together with SM), contributed to organizing field work and quality checks, contributed to the manuscript.

## Pre-publication history

The pre-publication history for this paper can be accessed here:



## Figures and Tables

**Table 1:** Description of cancer cases and controls and their potential as risk factors for colon cancer.

		No. cases^1^	No. controls^1^	Adj. OR (95% CI)
Age (continuous)	per year	354	1422	1.64 (1.52–1.77)
				
Age	< 50 years	47	249	referent
	50+ years	307	1173	4.38 (1.41–13.62)
				
Education	Lower education	279	1014	referent
	University level	64	394	0.64 (0.45–0.91)
				
Age at menarche (cont)	per year	344	1396	1.04 (0.95–1.14)
				
Age at menarche	< 13 years	148	683	referent
	13+ years	196	713	1.16 (0.87–1.55)
				
Pregnancy	Never pregnant	32	104	referent
	Ever pregnant	318	1300	0.85 (0.50–1.45)
				
Number of children (cont)	per child	318	1286	0.75 (0.64–0.89)
				
Age at first live birth (cont)	per birth	316	1274	1.00 (0.96–1.04)
				
Age at first live birth	< = 22 years	140	624	referent
	> 22 years	176	650	1.15 (0.84–1.57)
				
Breast-feeding	never	193	525	referent
	ever	157	878	0.31 (0.23–0.43)
				
OC use	never	144	484	referent
	ever	210	933	0.64 (0.46–0.89)
				
Family history of colon cancer	No	192	656	referent
	Yes	68	54	3.40 (2.08–5.56)
				
BMI (cont)		348	1406	0.96 (0.93–0.99)
				
Body mass index	< 25	135	505	referent
	25+	213	901	0.91 (0.68–1.23)

^1 ^Differences in numbers of cases/controls across variables are due to missing information

Conditional logistic regression: Age-adjusted odds ratios (OR) and 95% confidence intervals (CI).

**Table 2:** Risk of colon cancer and HRT use: Ever-use of HRT vs. never-use.

	**e-case^1^**	**e-ctrl.^1^**	**Adj. OR (95% CI)**
**Ever HRT**	150	562	0.97 (0.71–1.32)
**Duration of use^2,3^**			
1–4 yrs	22	130	0.65 (0.37–1.16)
5–9 yrs	41	180	1.06 (0.67–1.68)
10+ yrs	45	175	0.87 (0.53–1.41)
			
**Time since first use^2,3^**			
1–4 yrs	41	148	1.04 (0.66–1.66)
5–9 yrs	46	193	0.99 (0.64–1.52)
10–14 yrs	36	133	1.04 (0.65–1.69)
15+ yrs	27	88	0.79 (0.45–1.39)
			
**Time since last use^2,3^**			
< 1 yr/current use	136	496	1.00 (0.74–1.38)
1–2 yrs	8	43	0.72 (0.31–1.69)
3–4 yrs	5	22	0.85 (0.29–2.50)
5+ yrs	1	1	n.d.^4^

^1 ^e-case, e-ctrl = number of observations for exposed cases or exposed controls respectively.

^2 ^time variables were rounded

^3 ^no significant trend with increasing time

^4 ^n.d. = no data

Conditional logistic regression analysis [odds ratios (OR) and 95% confidence intervals]; adjustment for BMI, family history of colon cancer, childbearing history, age at first live birth, duration of breast-feeding, age at menarche, ever OC use, education. Index dates 0.5 = exposure information was not considered 0.5 years prior to cancer diagnosis.

**Table 3:** Risk of colon cancer associated with different categories of HRT formulation or administration.

	**e-case/e-ctrl**	**Adj. OR (95% CI)**
**Route of administration**		
ORAL		
Ever	99/381	0.76 (0.54–1.08)
1–4 years	17/108	0.54 (0.30–1.00)
5–9 years	27/125	0.61 (0.36–1.04)
10+ years	38/103	1.14 (0.68–1.92)
		
TRANSDERMAL		
Ever	24/99	1.17 (0.63–2.16)
1–4 years	6/29	0.98 (0.34–2.86)
5–9 years	5/32	0.87 (0.30–2.51)
10+ years	10/27	2.16 (0.73–6.36)
		
OTHER		
Ever	11/48	0.96 (0.40–2.31)
1–4 years	1/11	n.d.
5–9 years	2/11	n.d.
10+ years	5/19	1.15 (0.30–4.35)
		
**Form of combination**		
SEQUENTIAL FORMULATIONS		
Ever	46/191	0.80 (0.51–1.20)
1–4 years	5/49	0.47 (0.17–1.31)
5–9 years	14/66	0.66 (0.32–1.35)
10+ years	22/50	1.45 (0.70–3.04)
		
CONTINUOUS-COMBINED		
Ever	40/176	0.80 (0.50–1.29)
1–4 years	8/53	0.66 (0.26–1.66)
5–9 years	12/52	0.56 (0.25–1.20)
10+ years	9/47	0.65 (0.26–1.67)
		
ANY COMBINATION		
Ever	78/324	0.85 (0.59–1.21)
1–4 years	13/94	0.56 (0.28–1.12)
5–9 years	23/104	0.71 (0.40–1.24)
10+ years	28/83	1.21 (0.67–2.21)
		
**CEE/MPA combinations**		
CEE + MPA		
Ever	3/27	n.d
1–4 years	0/9	n.d.
5–9 years	1/7	n.d.
10+ years	1/10	n.d.
		
CEE or MPA		
Ever	42/142	0.64 (0.38–1.10)
1–4 years	6/36	0.44 (0.16–1.20)
5–9 years	13/46	0.63 (0.26–1.52)
10+ years	20/45	1.10 (0.49–2.47)
		
No CEE, no MPA		
Ever	80/306	0.95 (0.66–1.37)
1–4 years	19/89	0.87 (0.48–1.59)
5–9 years	21/104	0.78 (0.44–1.38)
10+ years	21/73	1.11 (0.58–2.13)
		
**Formulation content**		
**ONLY – HRTS**		
ESTROGEN ONLY		
Ever	55/189	0.81 (0.51–1.28)
1–4 years	8/46	0.50 (0.20–1.24)
5–9 years	14/66	0.98 (0.47–2.06)
10+ years	25/60	1.07 (0.52–2.19)
		
PROGESTAGEN ONLY		
Ever	9/35	0.48 (0.17–1.37)
1–4 years	1/10	n.d.
5–9 years	2/9	n.d.
10+ years	6/12	0.57 (0.13–2.48)
		
**Estrogen type**		
ESTRADIOL (E2)		
Ever	80/350	0.86 (0.60–1.22)
1–4 years	16/99	0.73 (0.39–1.36)
5–9 years	22/112	0.89 (0.52–1.54)
10+ years	28/92	1.10 (0.62–1.91)
		
CONJUGATED EQUINE ESTROGENS (CEE)		
Ever	40/141	0.70 (0.41–1.19)
1–4 years	5/35	0.44 (0.16–1.25)
5–9 years	12/47	0.61 (0.25–1.49)
10+ years	19/49	1.30 (0.57–2.73)
		
**Progestin type**		
NORETISTERONE ACETATE (NETA)		
Ever	42/193	0.78 (0.48–1.25)
1–4 years	7/57	0.54 (0.20–1.44)
5–9 years	13/61	0.73 (0.35–1.53)
10+ years	14/49	0.90 (0.42–1.94)
		
LNG		
Ever	22/83	0.88 (0.46–1.68)
1–4 years	2/21	0.45 (0.10–2.08)
5–9 years	6/35	0.53 (0.19–1.49)
10+ years	9/19	1.64 (0.51–5.30)
		
MEDROXYPROGESTERONE ACETATE (MPA)		
Ever	8/55	0.42 (0.16–1.10)
1–4 years	1/19	n.d.
5–9 years	3/13	n.d.
10+ years	3/16	n.d.
		
MEDROGESTONE		
Ever	18/65	0.87 (0.43–1.73)
1–4 years	3/17	n.d.
5–9 years	4/23	n.d.
10+ years	10/19	1.81 (0.62–5.28)
		
TIBOLONE		
Ever	5/14	0.91 (0.26–3.23)
1–4 years	2/6	n.d.
5–9 years	0/3	n.d.
10+ years	3/4	n.d.

The categories are not mutually exclusive – (see text). Ever use and three categories of duration of use in years are presented.

Conditional logistic regression: Odds ratios (OR) and 95% confidence intervals (95% CI); adjusted for age and region. No OR was calculated if the frequency of exposed cases or controls was fewer than 5 women. The only index date shown in this table is 0.5 years.

e-case/e-ctrl = number of exposed cases and controls, respectively
